# Identifying Diagnostic Thresholds for the Assessment of Cerebrovascular Reserve Impairment in Moyamoya Disease: CO_2_-Triggered BOLD-MRI and [^15^O]water-PET with Acetazolamide Challenge

**DOI:** 10.3390/brainsci16080888

**Published:** 2026-08-20

**Authors:** Patrick Haas, Lucas Moritz Wiggenhauser, Johannes Spang, Leonie Zerweck, Christian Volz, Marcos Tatagiba, Nadia Khan, Philipp T. Meyer, Till-Karsten Hauser, Constantin Roder

**Affiliations:** 1 Department of Neurosurgery and Moyamoya Center, University of Tübingen, Hoppe-Seyler-Straße 3, 72076 Tübingen, Germanymarcos.tatagiba@med.uni-tuebingen.de (M.T.);; 2 Department of Neuroradiology, University of Tübingen, Hoppe-Seyler-Straße 3, 72076 Tübingen, Germanytill-karsten.hauser@med.uni-tuebingen.de (T.-K.H.); 3 Department of Nuclear Medicine, Medical Center—University of Freiburg, Faculty of Medicine, University of Freiburg, Hugstetter Str. 55, 79106 Freiburg, Germany; 4 Moyamoya Center, University Children’s Hospital Zurich, University of Zurich, Steinwiesstrasse 75, 8032 Zurich, Switzerland

**Keywords:** cerebral revascularization, Moyamoya disease, cerebrovascular reserve, EC-IC Bypass

## Abstract

**Highlights:**

**What are the main findings?**

- Breath-hold BOLD MRI showed a strong correlation with acetazolamide-challenged [^15^O]water-PET for assessing cerebrovascular reserve in adults with Moyamoya disease.
- Cerebellum-normalized BOLD signal changes below 30% identified impaired reserve, whereas values below 60% provided a sensitive screening threshold.

**What are the implications of the main findings?**

- Breath-hold BOLD MRI may serve as an accessible triage tool to identify patients who require confirmatory PET-ACZ assessment.
- The proposed thresholds may support more efficient clinical decision-making, but prospective external validation is required before routine clinical implementation.

**Abstract:**

Background: Assessment of cerebrovascular reserve capacity (CVRC) is central to treatment planning in adult Moyamoya disease. [^15^O]-water-PET with acetazolamide challenge (PET-ACZ) serves as the reference standard but is resource-intensive and not universally available, whereas breathhold BOLD MRI (bh-fMRI) may offer a practical noninvasive alternative for screening and triage. Methods: This retrospective diagnostic accuracy study compared bh-fMRI and PET-ACZ in 67 adult MMD patients (1072 ROIs across 402 supratentorial vascular territories) to determine correlation and clinically useful MRI thresholds for impaired cerebrovascular reserve. Data were analyzed using Pearson’s correlation and ROC curves (STARD-compliant). Cerebellum-normalized percent signal change (CN-PSC) was referenced against PET-ACZ < 66% for pathological CVRC. Results: Supratentorial territories showed strong correlation (r = 0.71, 95%CI 0.62–0.80). Optimal diagnostic threshold was <28.2% CN-PSC on bh-fMRI (Youden index; sensitivity 83.8%, specificity 80.9%, AUROC = 0.87); screening threshold < 60% CN-PSC yielded sensitivity 94.6% and NPV 91.1%. No patient factors (age, Suzuki stage, etc.) influenced agreement (univariate/multivariate analyses). Conclusions: Bh-fMRI supplements PET-ACZ as a screening and diagnostic tool for cerebrovascular reserve impairment in adult MMD. PET remains the reference standard for confirmation, especially in intermediate or discordant cases, and the proposed thresholds (<30% CN-PSC: impaired CVRC; 30–60%: confirm with PET-ACZ; >60%: monitor) require prospective validation before routine decision-making use.

## 1. Introduction

Moyamoya disease (MMD) is characterized by steno-occlusive changes in the distal carotid arteries and their branches, leading to reduced blood flow and the development of compensatory collaterals. This collateral network, which resembles a “puff of smoke” on angiography may result in hemorrhagic infarctions from fragile vessels, ischemic strokes or chronic brain atrophy due to hypoperfusion [1,2,3]. Quantifying hypoperfused brain regions is crucial for indicating bypass surgery [4]. A key feature of MMD is neurovascular uncoupling, where the cerebral vascular system fails to regulate flow resistance in response to CO_2_ levels, making local cerebral blood flow (CBF) in affected areas reliant on blood pressure. This may lead to transient ischemic attacks or ischemic stroke, especially when the so-called “steal phenomenon” occurs [4]. Evaluation of the cerebral vascular reserve capacity (CVRC) is critical for planning revascularization surgery, as it reflects how well the brain can compensate for CBF changes in response to vasodilating stimuli like acetazolamide (ACZ) [5]. Imaging techniques, such as [^15^O]water positron emission tomography (PET) and single-photon emission computed tomography (SPECT) are used to measure CBF and CVRC, with PET being the reference standard due to its higher sensitivity [6,7,8,9,10]. However, high costs and limited availability reduce the use of [^15^O]water -PET/CT [11]. Blood oxygen level-dependent (BOLD) fMRI is an emerging alternative, offering another noninvasive way to assess cerebral vasoreactivity (CVR), a surrogate of CVRC [4,5,12,13,14,15,16]. Hypercapnia induced by breathholding maneuvers causes cerebral vasodilation (breathholding fMRI (bh-fMRI)). CVR is then determined by the increase in the BOLD signal. Prior research has demonstrated the reliability of bh-fMRI and good correlation between bh-fMRI and PET-ACZ in small data sets [13,14]. However, there are currently no practical CVR thresholds for the clinical triage of adult MMD patients. The primary endpoint of this study was the diagnostic agreement between bh-fMRI and PET-ACZ for assessment of cerebrovascular reserve capacity (CVRC), quantified as the Pearson correlation coefficient (r) and the area under the receiver operating characteristic curve (AUROC) of cerebellum-normalized percent signal change (CN-PSC) on bh-fMRI against the PET-ACZ reference standard (pathological CVRC defined as CN-PSC < 66%). Secondary endpoints included: (1) determination of an optimal diagnostic bh-fMRI threshold using the maximum Youden index; (2) determination of a screening bh-fMRI threshold optimized for sensitivity ≥ 95% and negative predictive value ≥ 90%; (3) evaluation of patient-specific and methodological factors influencing bh-fMRI–PET-ACZ agreement by univariate and multivariate analyses; and (4) diagnostic accuracy metrics including sensitivity, specificity, positive and negative predictive values, likelihood ratios (LR+ and LR−), and effect size (Cramér’s V).

## 2. Methods

### 2.1. MMD Cohort and Definition of Regions of Interest (ROIs)

**Figure 1:**
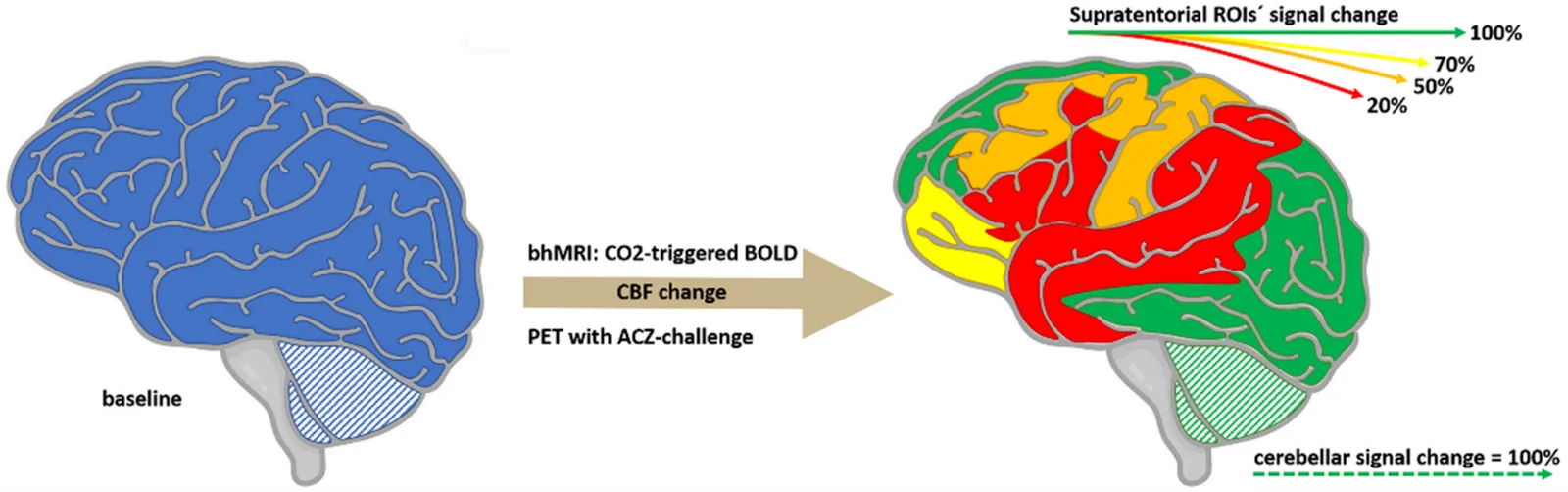
Principle of functional imaging with PET-ACZ and bh-fMRI for the detection of supratentorial signal changes in cerebral blood flow (CBF) is illustrated. The cerebellum serves as an intraindividual reference, postulating that there are no relevant CBF impairments due to MMD.

All MMD patients with concomitant bh-fMRI and PET-ACZ imaging treated at our center between August 2016 and August 2022 were included. PET-ACZ and bh-fMRI results were assigned to a total of 20 regions of interest (ROI), by using the automated anatomical atlas 3 (AAL3) as a standardized neuroanatomy atlas [17]. The ROIs were divided for each hemisphere into the vascular territories of the anterior cerebral artery (ACA, 2 ROIs), middle cerebral artery (MCA, 4 ROIs) and posterior cerebral artery (PCA, 2 ROIs), as well as the cerebellum (2 ROIs) as an intraindividual reference (Figure 1). The respective ROI with the lowest CVR was then decisive for the entire corresponding vascular territory. The choice of the cerebellum as a reference is justified as MMD does not affect the basilar or vertebral arteries. In order to avoid overemphasizing the effects of crossed cerebellar diaschisis seen in some patients, the cerebellar ROI with the strongest signal peak was chosen as the intraindividual reference instead of the mean cerebellar signal peak (cerebellum-normalized percent signal change (CN-PSC)).

### 2.2. Reference Standard: PET with Acetazolamide Challenge

**Figure 2:**
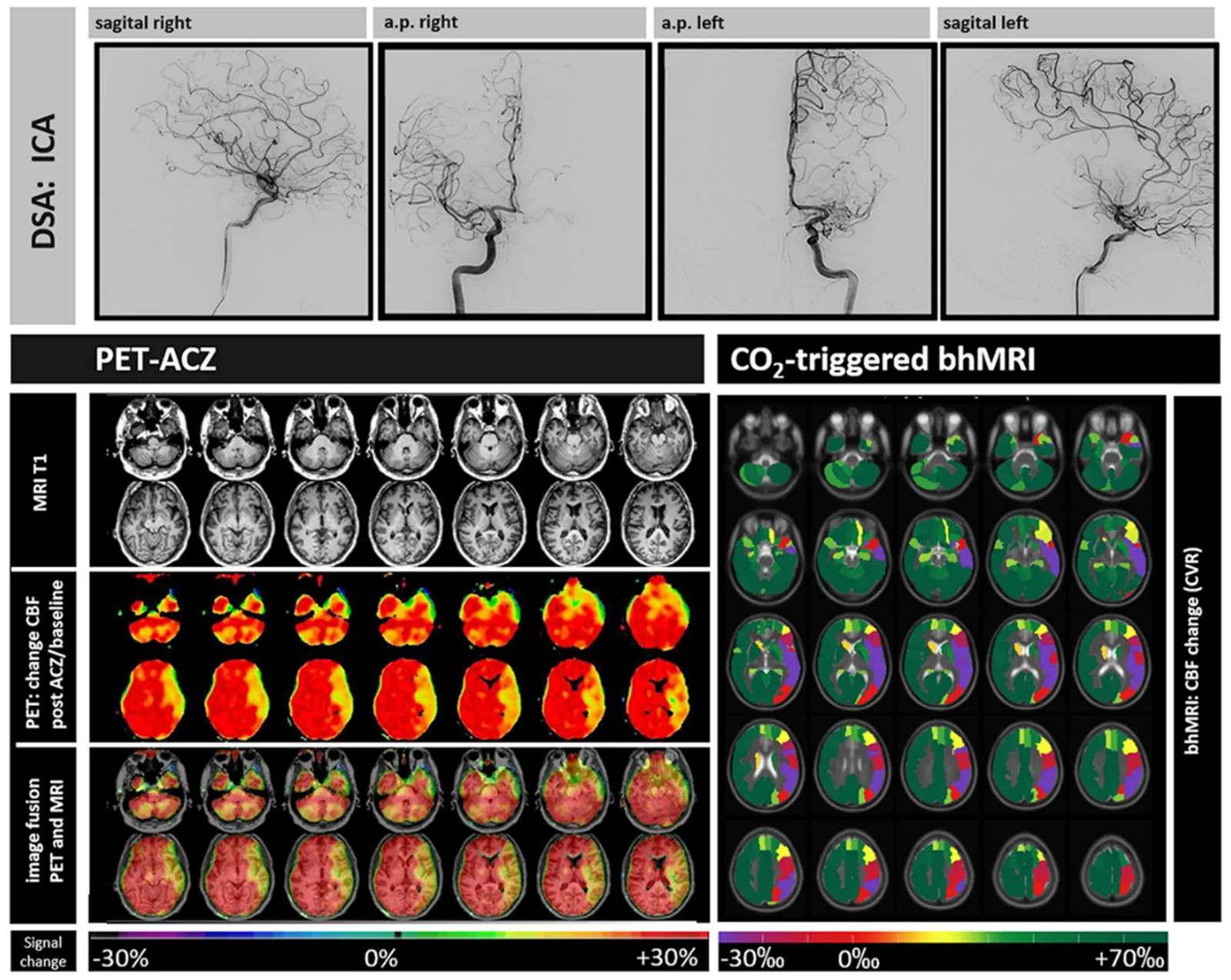
57-year-old male patient with unilateral MMD and occlusion of the left MCA with a rete mirabile as demonstrated in the DSA (upper row). There are already spontaneous leptomeningeal collaterals into the left MCA territory via the ipsilateral ACA and PCA. bh-fMRI shows a significant reduction (steal phenomenon) in cerebral vasoreactivity (CVR) in the left middle cerebral artery (MCA) territory. This finding is verified by the complementary positron emission tomography with acetazolamide challenge (PET-ACZ) (bottom row, left), which reveals analogous results. Note: The absolute signal changes are shown (PET-ACZ voxel-based, bh-fMRI ROI-based) whereas for the calculations described above, normalization was performed relative to the cerebellum reference (CN-PSC).

PET scans were acquired on a Vereos digital PET/CT system (Philips Healthcare, Amsterdam, The Netherlands) and analyzed as previously described [14,18,19]. In short, two 4 min PET scans each (bolus injection of 291.2 ± 23.7 MBq [^15^O]water per scan) were performed before (baseline) and after a 5 min infusion (10 mL) of a standard dose of 1000 mg ACZ (post-ACZ). The interscan interval was 10 min, with the ACZ infusion being started immediately after the second baseline scan (the first post-ACZ scan started 10 min after start of the ACZ infusion). CVRC maps were calculated as voxel-wise signal change [%] between the averaged baseline and post-ACZ maps, which were derived by integrating the dynamic PET images over 60 s after the arrival of the tracer in the individual patient’s brain [10]. CRP maps were displayed in 32 transaxial planes covering the entire brain using a harmonized “rainbow” color scale. The color scale was thresholded symmetrically around zero (i.e., max/min set to ±[mean value + 2 standard deviations of CVRC in cerebellar and PCA territories]) and customized such that the zero transition (i.e., watershed in the case of CBF steal) was marked by three black bins within the 256-bin color scale (Figure 2). A CN-PSC of <66% on PET-ACZ was defined as the threshold for a pathological reduction in CVR based on our clinical experience. The investigators of the reference standard PET-ACZ were blinded to the results of the index test with bh-fMRI.

### 2.3. Index Test: CO_2_-Triggered BOLD MRI

A 3T MR scanner (Magnetom Skyra, Vida fit, Siemens, Erlangen, Germany) with a 20-channel head coil was used to perform a T2*-weighted EPI sequence. Imaging parameters were the following: the repetition time (TR) was 3000 ms, the echo time (TE) was 3~ms, the matrix size was 96 × 96, the slice thickness was 3~mm, the number of slices was 34, and the field of view (FOV) was 256. The breathhold paradigm consisted of 1 min of normal breathing and was followed by 7 cycles of 9 s of breathholding in partial expiration and 60 s of regular breathing. Breathing instructions were presented visually using a mirror and a wall-mounted display. Data processing was performed as described previously (Figure 2) [12]. In short, DICOM images were exported to a dedicated workstation. After conversion to nifti format, preprocessing consisted of slice timing correction, realignment, unwarping and normalization to MNI space. All preprocessing steps were calculated using SPM12 (Wellcome Center for Human Neuroimaging, London, UK) running on MATLAB R2024B (The MathWorks Inc., Natick, MA, USA). After normalization, the breathhold cycles were averaged, detrended and mean percentage signal change was calculated for each of the 120 VOIS of the AAL atlas (AAL3V1) [20]. The minimum signal change in the ROIs of each supratentorial vascular territory was set in relation to the maximum cerebellar signal change as a reference (CN-PSC). The investigators of the index test bh-fMRI were blinded to the results of the reference standard with bh-fMRI.

### 2.4. Data Processing, Analysis and Statistics

The diagnostic study was planned and analyzed in accordance with the Standards for the Reporting of Diagnostics Accuracy Studies guidelines (STARD statements). All data were stored in a custom database (REDCap) and statistically analyzed (JMP, Version 17, SAS Institute, Cary, NC, USA). Concomitant bh-fMRI and PET-ACZ images were subjected to Pearson correlation analyses for evaluation of data association. Pearson’s r > 0.5 was considered a moderate correlation, r > 0.7 a strong and r > 0.85 a very strong correlation. Correlation analysis was complemented by linear regression analysis for evaluation of agreement. Subsequently, PET-ACZ was compared with bh-fMRI by analyzing the area under the curve of a receiver operating characteristic (AUROC). Two separate threshold values for bh-fMRI were determined: (1) The maximum of Youdens J was used to calculate the threshold as a compromise between sufficient sensitivity (SENS) and specificity (SPEC) for bh-fMRI as a diagnostic tool. (2) For bh-fMRI as a screening tool, an ROC analysis was used to optimize the threshold value under the constraints of high SENS ≥ 95% and high negative predictive value (NPV) ≥ 90%. The SENS, SPEC and NPV for these threshold values were calculated by contingency analysis. Likelihood ratios (LR) for positive predictive values (LR+) and negative predictive values (LR−) with 95% confidence intervals (95%CI) were calculated as the single measure of the accuracy of the diagnostic test (bh-fMRI). LR+ is defined as the probability that an MMD patient with impaired CVRC on PET-ACZ will have an impaired CVR on bh-fMRI divided by the probability that an MMD patient without CVRC impairment will have an impaired CVR on bh-fMRI. LR− is the probability that an MMD patient with impaired CVRC on PET-ACZ will have a non-impaired CVR on bh-fMRI divided by the probability that an MMD patient without restricted CVRC will have a non-impaired CVR on bh-fMRI. Contingency tables were analyzed with Fisher’s exact test. Effect size for Chi-square tests were calculated using Cramer’s V. A V-Value ≥ 0.3 was considered a moderate effect, V ≥ 0.5 was considered a strong effect. Homogeneity of variance was assessed using Levene’s test, followed by pooled/unpooled *t*-tests, as appropriate, for univariate testing of potential factors affecting the correlation between bh-fMRI and PET-ACZ. *p*-values < 0.05 were considered significant. After univariate testing nominal logistic regression models were used for multivariate analysis. Odds ratios (OR) with 95%CI were calculated as a measure of the strength of association. To account for within-patient correlation across ROI-level measurements, we fitted a linear mixed-effects model with patient ID as a random intercept. PET CN-PSC was modeled as a function of bh-fMRI CN-PSC, thereby accounting for the non-independence of multiple ROIs within the same patient.

### 2.5. Standard Protocol Approvals, Registrations, and Patient Consents

All procedures performed were in accordance with the ethical standards of the institutional research committee and with the 1964 Helsinki declaration and its later amendments or comparable ethics standards. Approval of the local ethics committee was obtained (084/2023BO2).

## 3. Results

### 3.1. Participants

A total of 67 concomitant patient data sets were included, comprising both bh-fMRI and PET-ACZ. The study examined a total of 402 supratentorial vascular territories (ACA, MCA, PCA) in *n* = 65 individuals diagnosed with MMD. Two patients received follow-up examinations 26 and 65 months apart from the initial examination. A total of 1340 ROIs were included in the correlation analysis. The mean time interval between bh-fMRI and PET-ACZ was 60 days ± 56. The MMD patient collective showed a typical age and gender distribution with a mean age of 41.0 ±13.3 years and female predominance of 2.8:1. A large proportion of the patients were of Caucasian origin, the initial MMD manifestation was mainly characterized by transient and manifest ischemic events (72.3%). Table 1 presents a summary of the characteristics of the patients.

### 3.2. PET Results and Correlation with bh-fMRI

On PET, n = 484 ROIs (22.6%) and correspondingly n = 167 (41.5%) vascular territories were evaluated as having a reduced CVRC with less than 66% CN-PSC. A relevant CVRC reduction was thus detected in n = 58 (86.6%) of all patient data sets in one or more vascular territories. Mean Pearson correlation for supratentorial vascular territories (ACA, MCA, PCA) between PET and MRI signal change was r = 0.71 (95% CI 0.62–0.80, *p* < 0.001), indicating a significant linear correlation. In the mixed-effects model, bh-fMRI CN-PSC was strongly associated with PET CN-PSC (fixed effect estimate 0.275, standard error 0.021, *p* < 0.0001; 95% CI 0.233–0.317). The intercept was 0.561 (standard error 0.037, *p* < 0.0001; 95% CI 0.488–0.634). The random intercept for patient ID indicated substantial within-patient clustering of ROI-level data.

### 3.3. Test Accuracy and Precision

#### 3.3.1. bh-fMRI as Screening Tool

The CVR thresholds of bh-fMRI, which is intended to function as a screening tool with high SENS and NPV, were determined in a stepwise approach as described above. Using a threshold of <60% CN-PSC for impaired CVR signal increase on bh-fMRI, the test achieved an AUROC of 0.78 (*p* < 0.0001), with 94.6% SENS and a 91.1% NPV. Concordance with PET-ACZ was moderate (Cramér’s V = 0.41, *p* < 0.0001). The LR− of 0.14 (95% CI 0.07–0.27) indicates that a negative bh-fMRI finding meaningfully reduces the posttest likelihood of impaired CVRC on PET-ACZ.

Accordingly, when bh-fMRI is used as a screening tool, a CVR ≥ 60% may support surveillance with follow-up bh-fMRI rather than immediate PET-ACZ, as long as clinical and imaging findings remain otherwise unremarkable. In contrast, a CVR < 60% should be considered an indication for further clarification by PET-ACZ.

#### 3.3.2. bh-fMRI as Diagnostic Tool

**Figure 3:**
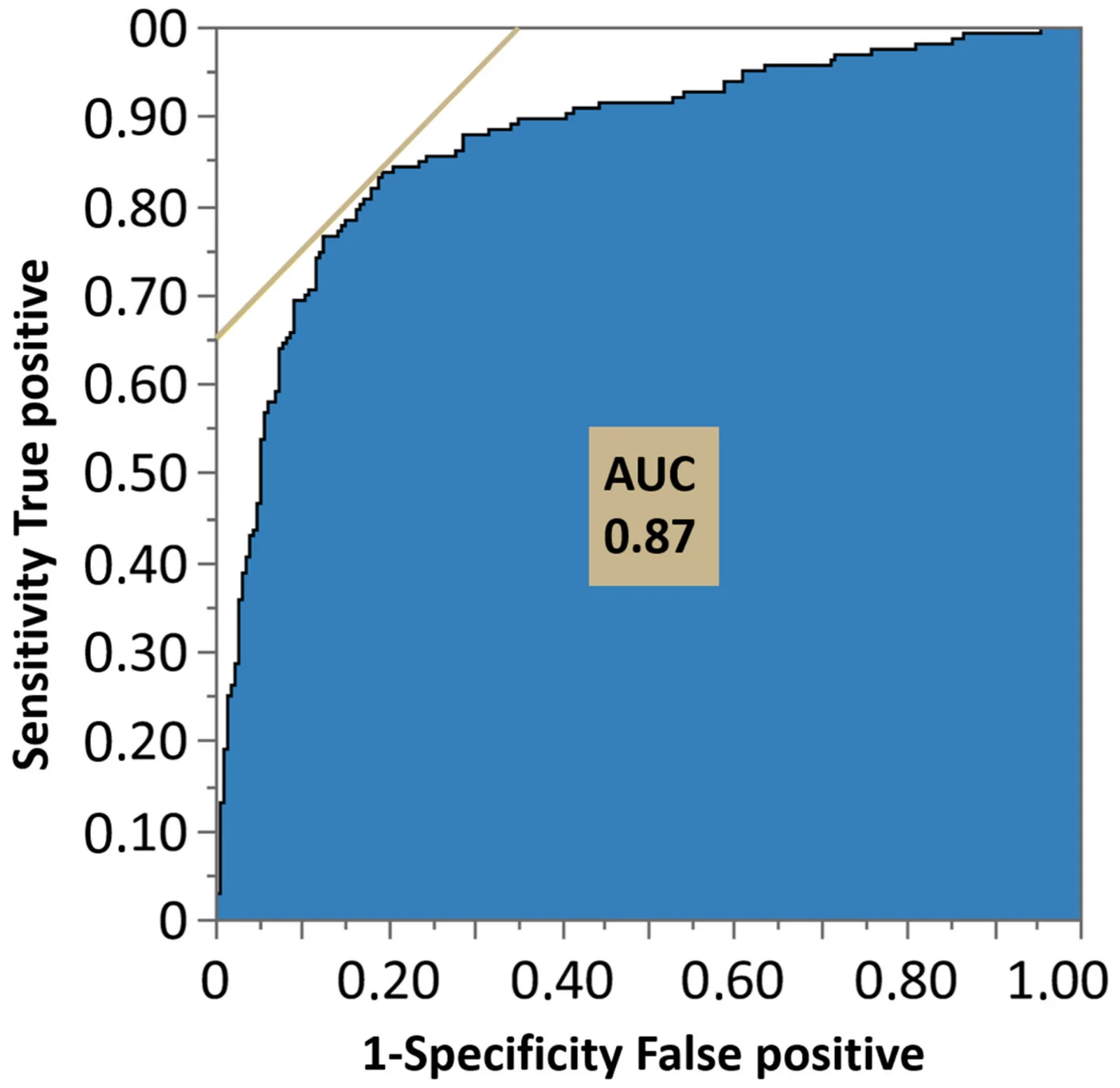
Receiver operating characteristics (ROC) curve with an AUC of 0.87. The calculation of the Youden index resulted in a cutoff value of a pathologically reduced CVR at <28.2% signal increase for bh-fMRI. For this threshold value, a SENS of 83.8% and a SPEC of 80.9% could be calculated for the bh-fMRI.

**Figure 4:**
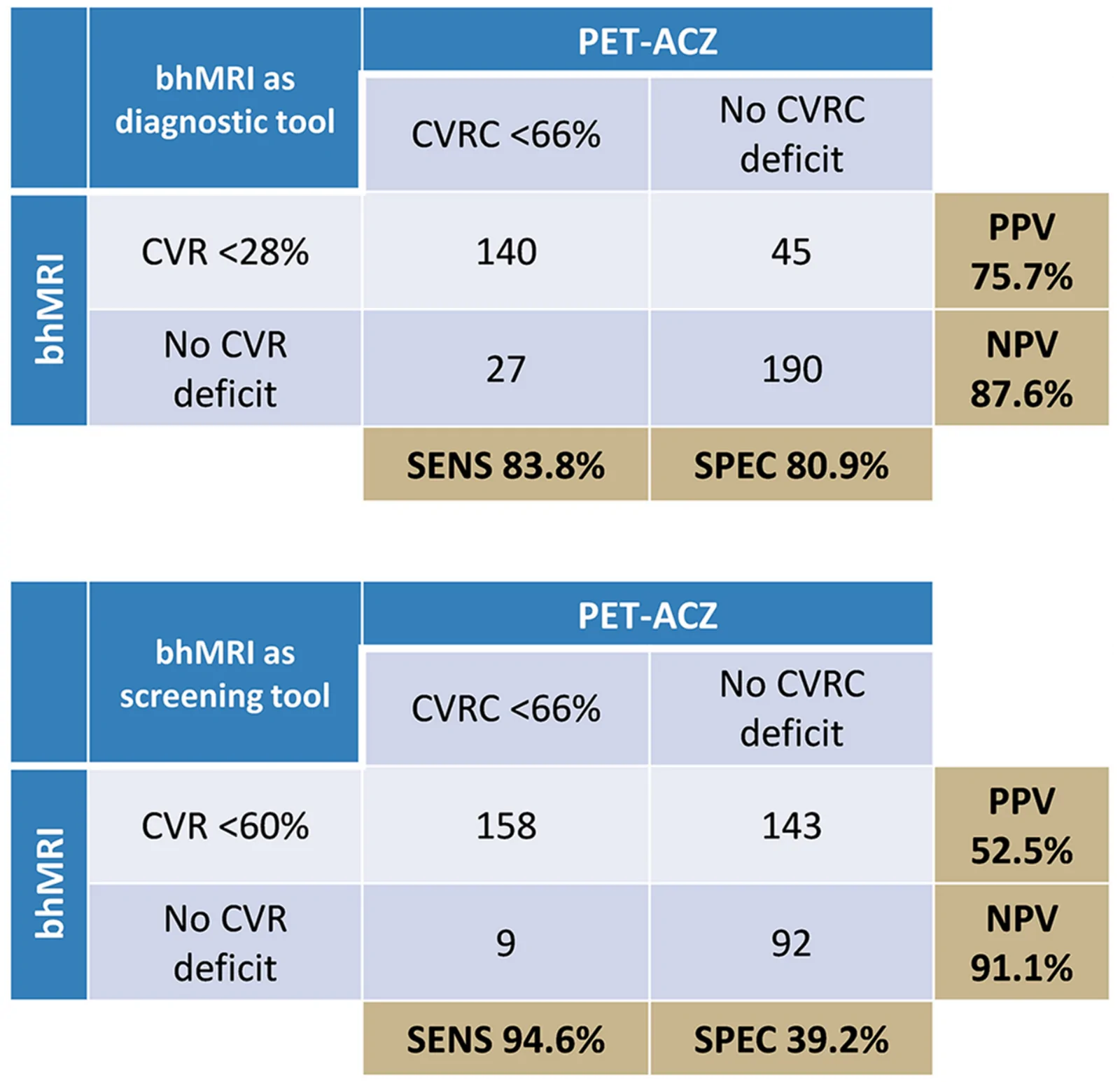
Contingency tables for the two applications of bh-fMRI as a diagnostic tool (upper table) with the optimal compromise of sensitivity (SENS) and specificity (SPEC) determined using the Youden index, and for use as a screening tool with high SENS and negative predictive value (NPV) (lower table).

The highest Youden index was seen for a threshold value of a pathologically reduced CVR at <28.2% CN-PSC on bh-fMRI for the global vascular ROIs of the ACA, MCA and PCA territories. An AUROC of 0.87 could be calculated for this threshold value (*p* < 0.0001) (Figure 3). This resulted in a SENS of 83.8% and a SPEC of 80.9% with strong correlation (*p* < 0.0001, Cramer’s V = 0.66) (Figure 4). The positive likelihood ratio (LR+) for impaired CVR on bh-fMRI with a concomitant test result of impaired CVRC on PET-ACZ was 4.35 (95%CI 3.31–5.70), resulting in an MMD patient with impaired CVRC being 4.35 times as likely to test positive (impaired CVRC) on bh-fMRI compared to an MMD patient without impaired CVR on bh-fMRI. A pathological bh-fMRI with a CVR < 28.2% CN-PSC substantially increases the pretest probability of hemodynamically relevant CVRC restriction in PET-ACZ. In order to facilitate the implementation of the calculated CVR limit value in clinical routine, it was rounded to 30% CN-PSC for the following discussion.

### 3.4. Potential Factors Influencing the Correlation Between bh-fMRI and PET-ACZ

A number of patient-specific factors that could potentially influence bh-fMRI results were analyzed: age, sex, ethnicity, disease stage according to Suzuki classification, disease manifestation (ischemic stroke, hemorrhagic stroke, transitory ischemic attack (TIA), minor performance deficit, incidental finding) and vascular risk factors such as smoking, arterial hypertension, diabetes mellitus and increased body mass index (BMI). No significant predictors for the correlation strength between PET-ACZ and bh-fMRI (agreement in perfusion deficit) were identified, neither in univariate nor multivariate testing. The potential impact of method-dependent variables, including the timing of imaging and the interval between bh-fMRI and PET-ACZ, was also evaluated. However, these factors also did not significantly influence the observed correlation. No adverse events occurred during the performance of either the index test or the reference standard.

## 4. Discussion

This retrospective cross-sectional diagnostic accuracy study aimed to compare functional imaging with bh-fMRI with the reference standard PET-ACZ for the determination of CVRC in adult MMD patients within a substantial large data set. It also aimed to provide clinically relevant cutoff values for decision-making. Two distinct threshold values for bh-fMRI were established with associated test quality criteria for evaluating the validity. However, it is imperative to note that these findings are exclusively applicable to the retrospective data set and necessitate validation through prospective studies. The established limits might however already be applicable in clinical practice. They facilitate the interpretation of bh-fMRI results and provide treating physicians with additional information indicating PET-ACZ, which still is the gold standard in perfusion diagnostics for MMD. In consideration of these established thresholds, bh-fMRI may be utilized in conjunction with PET as a screening instrument. The potential of bh-fMRI as a diagnostic instrument in staging and monitoring throughout the course of the disease must first be investigated further using prospective data sets [16,21,22,23]. MRI might provide supplementary diagnostic insights through the identification of infarct regions, atrophy assessment, and even disease progression with contrast-enhanced vessel wall imaging (VW-CE) [24,25].

### 4.1. Implications of Threshold Values on bh-fMRI for Clinical Practice

A CVR threshold (to define an insufficient CVR compared to the cerebellum) of approximately less than 30% CN-PSC enables bh-fMRI to function as a valuable diagnostic tool, with a good SENS of 83.8% and SPEC of 80.9%. However, if only these bh-fMRI results were considered, 24.3% of patients (1-PPV) would still receive an erroneous bypass indication. Therefore, it is crucial to indicate revascularization surgery not only on bh-fMRI but to consider other factors such as, e.g., previous ischemic or hemorrhagic events, clinical symptoms and pathological concentric VW-CE as a sign of progressive disease [24,25]. PET-ACZ diagnostics remain the gold standard and should be performed to confirm the indication for revascularization surgery if any of the before-mentioned examination results are not conclusive. If the CVR signal change is between 30% and 60% CN-PSC, bh-fMRI has sufficient quality as a screening test for detection of possible tissue-at-risk with impaired CVR with a SENS of 94.6% and an NPV of 91.1%. But as these reliable results are seen within a screening test, a PET-ACZ should then be carried out as a diagnostic test for confirmation. In 35.6% of patients exhibiting such intermediate CVR reduction on bh-fMRI, there was no corroboration on PET-ACZ, suggesting the absence of inadequate CVRC. On the other hand, only 8.9% (1-NPV) of patients with negative bh-fMRI findings would be mistakenly not recommended for PET diagnostics. However, in a clinical setting, this figure is likely to be significantly lower, as the decision to perform PET-ACZ is not based solely on bh-fMRI results, but is made after reviewing all clinical, magnetic resonance imaging, and angiographic findings. Clinically, breathhold BOLD MRI may be used as a practical triage tool helping decision-making in adult MMD, with values below 30% CN-PSC suggesting pathologically reduced CVRC, intermediate values between 30% and 60% CN-PSC warranting confirmatory PET-ACZ, and values above 60% CN-PSC supporting surveillance with follow-up MRI in the absence of other concerning clinical or imaging findings. Consequently, PET can be employed in MMD in a more targeted and resource-efficient manner. It is also important to note that bh-fMRI allows for more frequent follow-up monitoring due to its accessibility compared to PET. Bh-fMRI’s capacity for longitudinal monitoring enables not only the identification of CVR alterations, but also other MMD-specific characteristics such as new ischemic lesions or VW-CE, thereby encompassing the dynamic nature of MMD in the decision-making process for revascularization. In conclusion, it can be stated that bh-fMRI has its place in the functional imaging of MMD alongside the gold standard PET-ACZ. It is recommended that both of these modalities might be considered as integral components of MMD diagnostics. However, it should be noted that they do not provide the sole indication for revascularization. The decision to proceed with revascularization is made on a multimodal, patient-specific basis, taking all imaging findings and clinical symptoms into account.

### 4.2. Principles of bh-fMRI and Its Limitations

Neurovascular coupling as a mechanism of cerebrovascular autoregulation represents a complex interaction of biochemical, electrophysiological, and cellular signaling pathways involving astrocytes, neurotransmitters such as glutamate, and vascular smooth muscle cells, among others [26,27]. Despite extensive research, this phenomenon remains incompletely understood. The BOLD signal, which correlates with neurovascular coupling, is influenced by several factors, including CBV, CBF, oxygen extraction rate, capillary diameter, and hematocrit [28].

The study design primarily examined the validity of bh-fMRI. Consequently, statements about diagnostic reliability cannot be derived and require a test–retest design. Furthermore, patient compliance, partial pressure of carbon dioxide (paCO_2_) and vital signs during the breathhold maneuver were not objectively recorded. The implementation of gas delivery systems capable of delivering reliable and repeatable vasoactive CO_2_ stimuli is proposed to serve as an effective solution, but also has several drawbacks and is more invasive than breathholding [29]. Potential influencing factors, such as neurological impairment or emotional stress with its vegetative components, tachycardia, increased high blood pressure, and hyperventilation with a corresponding influence on the acid–base homeostasis, might as well be considered. While standardized breathhold paradigms were employed in the present study design, the optimal duration and timing (end-expiration vs. end-inspiration) remain undetermined, whereas we assume and practice end-expiration tasks to be the superior method. The heterogeneous breathhold paradigms described in the literature present a significant challenge to the comparison of these results. However, it has been demonstrated that this parameter influences both interscan variability and the accuracy of the measurement [30,31]. Furthermore, there is a sigmoidal relationship between the BOLD signal and paCO_2_ [32]. However, for the purposes of this study design, a simplified linear relationship was assumed. This is demonstrated to be the case at least for a paCO_2_ (more precisely, partial end-tidal CO_2_ (petCO_2_)) in the range between 40 and 50 mmHg, with a good fit, especially in the presence of a neurovascular disease such as MMD, and can therefore be approximated for endogenous hypercapnia due to short-term apnea [33]. Further studies are required to ascertain whether the thresholds established herein can be applied to bh-fMRI with alternative breathhold paradigms. While bh-fMRI uses a relative signal change to determine CVR, PET already provides information about CBF at baseline. However, these baseline values were not taken into account in this study; only the signal change in PET due to ACZ challenge was compared. It is important to acknowledge that PET-ACZ thresholds for impaired CVRC vary considerably across centers and studies, ranging from absolute CBF increases of <10 mL/100 g/min to semiquatitative or relative CVR thresholds, with some groups defining pathology based on institutional norms or healthy control distributions [9,34]. The <66% CN-PSC threshold used in this study was derived from our institutional clinical experience and internal validation against clinical outcomes, but requires external validation in independent cohorts before generalization.

## 5. Conclusions

Both PET and bh-fMRI are instruments for assessing CVRC and CVR in adult patients with MMD, with PET representing the gold standard in functional imaging. After examining the value of bh-fMRI for screening and diagnostics, a diagnostic algorithm was proposed. In combination with PET imaging using acetazolamide challenge, bh-fMRI serves as a valuable supplementary diagnostic and screening tool. When clinical findings and conventional angiography are definitive, a compromised CVR, indicated by a CBF increase of approximately <30% compared to the cerebellum on bh-fMRI using the 9s breathhold paradigm, may suggest the need for revascularization with high sensitivity. In instances where the aforementioned clinical and imaging findings are discrepant from the results of bh-fMRI, or if the CVR reduction determined on the bh-fMRI falls within the borderline range of 30% to 60% CN-PSC, additional PET-ACZ imaging is recommended. However, these thresholds of the algorithm proposed here must first be validated prospectively. Nonetheless, the indication for revascularization remains multimodal: it is not solely based on comprehensive functional imaging, but also takes clinical manifestations into account, and should therefore invariably be made on an interdisciplinary and patient-specific basis.

## Figures and Tables

**Table 1:** Patient characteristics.

Item	mean ± SD or Count (%)
Age	41.0 years ± 13.3
Sex	
female	48 (73.8%)
male	17 (26.1%)
Ethnicity	
Caucasian	58 (89.2%)
Asian	7 (10.8%)
BMI	26.0 ± 5.0
Diabetes mellitus	7 (10.9%)
Smoking	19 (29.7%)
Initial MMD manifestation	
TIA	16 (24.6%)
Ischemic stroke	31 (47.7%)
Hemorrhagic stroke	4 (6.1%)
Minor deficit (impaired neurocognition or headache)	13 (20.0%)
Incidental	1 (1.5%)
MMD type	
Bilateral (right/left)	42 (64.6%)
Suzuki 1	3 (7.1%)/6 (14.3%)
Suzuki 2	10 (23.8%)/4 (9.5%)
Suzuki 3	10 (23.8%)/11 (26.2%)
Suzuki 4	8 (19.0%)/8 (19.0%)
Suzuki 5	7 (16.7%)/7 (16.7%)
Suzuki 6	4 (9.5%)/5 (11.9%)
Unilateral right	14 (21.5%)
Suzuki 1	0 (0%)
Suzuki 2	3 (23.1%)
Suzuki 3	7 (53.8%)
Suzuki 4	1 (7.7%)
Suzuki 5	2 (15.4%)
Suzuki 6	0 (0%)
Unilateral left	9 (13.9%)
Suzuki 1	0 (0%)
Suzuki 2	0 (0%)
Suzuki 3	6 (66.7%)
Suzuki 4	2 (22.2%)
Suzuki 5	0 (0%)
Suzuki 6	1 (11.1%)
Time delta MRI to PET (days)	60 ± 56

## Data Availability

The data presented in this study are available on request from the corresponding author due to ethical restrictions requiring approval by the relevant ethics committee.

## Abbreviations

The following abbreviations are used in this manuscript:

ACA	anterior cerebral artery
ACZ	acetazolamide
AUROC	area under the curve receiver operating characteristics
bh-fMRI	breathhold functional magnetic resonance imaging
BMI	body mass index
BOLD	blood-oxygenation-level dependent
CBF	cerebral blood flow
CN-PSC	cerebellum-normalized percent signal change
CVR	cerebral vasoreactivity
CVRC	cerebral vascular reserve capacity
EC-IC	extracranial–intracranial
FOV	field of view
ICA	internal carotid artery
LR	likelihood ratio
MCA	middle cerebral artery
MMD	Moyamoya disease
MRI	magnetic resonance imaging
NPV	negative predictive value
OR	odds ratio
paCO_2_	partial pressure of carbon dioxide
PCA	posterior cerebral artery
PET	positron emission tomography
petCO_2_	partial end-tidal carbon dioxide
PPV	positive predictive value
ROI	region of interest
SPECT	single-photon emission computed tomography
TE	echo time
TR	repetition time
VW-CE	contrast-enhancing vessel wall

## References

[1] Takeuchi K. (1957). Hypoplasia of bilateral internal carotid arteries. Brain Nerve.

[2] Suzuki J., Takaku A. (1969). Cerebrovascular “moyamoya” disease. Disease showing abnormal net-like vessels in base of brain. Arch. Neurol..

[3] Haas P., Debolski A., Bender B., Zerweck L., Ernemann U., Tatagiba M., Hauser T.K., Khan N., Roder C. (2025). Whole-brain volumetric analysis in adult Moyamoya patients reveals significant atrophy compared to healthy controls. Brain Commun..

[4] Pillai J.J., Mikulis D.J. (2015). Cerebrovascular reactivity mapping: An evolving standard for clinical functional imaging. Am. J. Neuroradiol..

[5] Lehman V.T., Cogswell P.M., Rinaldo L., Brinjikji W., Huston J., Klaas J.P., Lanzino G. (2019). Contemporary and emerging magnetic resonance imaging methods for evaluation of moyamoya disease. Neurosurg. Focus.

[6] Kazumata K., Tha K.K., Uchino H., Ito M., Nakayama N., Abumiya T. (2017). Mapping altered brain connectivity and its clinical associations in adult moyamoya disease: A resting-state functional MRI study. PLoS ONE.

[7] Li J., Jin M., Sun X., Li J., Liu Y., Xi Y., Wang Q., Zhao W., Huang Y. (2019). Imaging of Moyamoya Disease and Moyamoya Syndrome: Current Status. J. Comput. Assist. Tomogr..

[8] Schubert G.A., Weinmann C., Seiz M., Gerigk L., Weiss C., Horn P., Thomé C. (2009). Cerebrovascular insufficiency as the criterion for revascularization procedures in selected patients: A correlation study of xenon contrast-enhanced CT and PWI. Neurosurg. Rev..

[9] Acker G., Lange C., Schatka I., Pfeifer A., Czabanka M.A., Vajkoczy P., Buchert R. (2018). Brain Perfusion Imaging Under Acetazolamide Challenge for Detection of Impaired Cerebrovascular Reserve Capacity: Positive Findings with ^15^O-Water PET in Patients with Negative ^99m^Tc-HMPAO SPECT Findings. J. Nucl. Med..

[10] Arigoni M., Kneifel S., Fandino J., Khan N., Burger C., Buck A. (2000). Simplified quantitative determination of cerebral perfusion reserve with H_2_^15^O PET and acetazolamide. Eur. J. Nucl. Med..

[11] Liu P., Li Y., Pinho M., Park D.C., Welch B.G., Lu H. (2017). Cerebrovascular reactivity mapping without gas challenges. Neuroimage.

[12] Hauser T.-K., Seeger A., Bender B., Klose U., Thurow J., Ernemann U., Tatagiba M., Meyer P.T., Khan N., Roder C. (2019). Hypercapnic BOLD MRI compared to H_2_^15^O PET/CT for the hemodynamic evaluation of patients with Moyamoya Disease. NeuroImage Clin..

[13] Roder C., Klose U., Hurth H., Brendle C., Tatagiba M., Ernemann U., Khan N., Hauser T.K. (2021). Longitudinal Reproducibility of CO_2_-Triggered BOLD MRI for the Hemodynamic Evaluation of Adult Patients with Moyamoya Angiopathy. Cerebrovasc. Dis..

[14] Zerweck L., Hauser T.K., Roder C., Blazhenets G., Khan N., Ernemann U., Meyer P.T., Klose U. (2023). Evaluation of the cerebrovascular reactivity in patients with Moyamoya Angiopathy by use of breath-hold fMRI: Investigation of voxel-wise hemodynamic delay correction in comparison to [^15^O]water PET. Neuroradiology.

[15] Fierstra J., van Niftrik C., Warnock G., Wegener S., Piccirelli M., Pangalu A., Esposito G., Valavanis A., Buck A., Luft A. (2018). Staging Hemodynamic Failure With Blood Oxygen-Level-Dependent Functional Magnetic Resonance Imaging Cerebrovascular Reactivity: A Comparison Versus Gold Standard (^15^O-)H_2_O-Positron Emission Tomography. Stroke.

[16] Mikulis D.J., Krolczyk G., Desal H., Logan W., deVeber G., Dirks P., Tymianski M., Crawley A., Vesely A., Kassner A. (2005). Preoperative and postoperative mapping of cerebrovascular reactivity in moyamoya disease by using blood oxygen level—Dependent magnetic resonance imaging. J. Neurosurg..

[17] Rolls E.T., Huang C.-C., Lin C.-P., Feng J., Joliot M. (2020). Automated anatomical labelling atlas 3. NeuroImage.

[18] Zerweck L., Roder C., Hauser T.-K., Thurow J., Mengel A., Tatagiba M., Khan N., Meyer P.T., Ernemann U., Klose U. (2021). Hemodynamic evaluation of patients with Moyamoya Angiopathy: Comparison of resting-state fMRI to breath-hold fMRI and [^15^O]water PET. Neuroradiology.

[19] Zerweck L., Klose U., Roder C., Staber D., Renger E., Blazhenets G., Grundmann-Hauser K., Meyer P.T., Ernemann U., Hauser T.K. (2025). Measuring cerebrovascular reactivity with breath-hold fMRI in patients with Moyamoya angiopathy: MR perfusion based delay correction significantly improves agreement to [^15^O]water PET. Neuroradiology.

[20] Tzourio-Mazoyer N., Landeau B., Papathanassiou D., Crivello F., Etard O., Delcroix N., Mazoyer B., Joliot M. (2002). Automated anatomical labeling of activations in SPM using a macroscopic anatomical parcellation of the MNI MRI single-subject brain. Neuroimage.

[21] Rosen C., McKetton L., Russell J., Sam K., Poublanc J., Crawley A., Han J.S., Sobczyk O., Duffin J., Mandell D.M. (2018). Long-term changes in cerebrovascular reactivity following EC-IC bypass for intracranial steno-occlusive disease. J. Clin. Neurosci. Off. J. Neurosurg. Soc. Australas..

[22] Sam K., Poublanc J., Sobczyk O., Han J.S., Battisti-Charbonney A., Mandell D.M., Tymianski M., Crawley A.P., Fisher J.A., Mikulis D.J. (2015). Assessing the effect of unilateral cerebral revascularisation on the vascular reactivity of the non-intervened hemisphere: A retrospective observational study. BMJ Open.

[23] Yun T.J., Cheon J.E., Na D.G., Kim W.S., Kim I.O., Chang K.H., Yeon K.M., Song I.C., Wang K.C. (2009). Childhood moyamoya disease: Quantitative evaluation of perfusion MR imaging—Correlation with clinical outcome after revascularization surgery. Radiology.

[24] Shih-Yüng Wang S., Hauser T.K., Haas P., Tellermann J., Hurth H., Ernemann U., Tatagiba M., Bender B., Khan N., Roder C. (2024). Intensity Score of Vessel Wall Contrast Enhancement in MRI Allows Prediction of Disease Progression in Moyamoya Angiopathy. Neurosurgery.

[25] Roder C., Hauser T.K., Ernemann U., Tatagiba M., Khan N., Bender B. (2019). Arterial wall contrast enhancement in progressive moyamoya disease. J. Neurosurg..

[26] Attwell D., Buchan A.M., Charpak S., Lauritzen M., Macvicar B.A., Newman E.A. (2010). Glial and neuronal control of brain blood flow. Nature.

[27] Koehler R.C., Roman R.J., Harder D.R. (2009). Astrocytes and the regulation of cerebral blood flow. Trends Neurosci..

[28] Blockley N.P., Griffeth V.E., Simon A.B., Buxton R.B. (2013). A review of calibrated blood oxygenation level-dependent (BOLD) methods for the measurement of task-induced changes in brain oxygen metabolism. NMR Biomed..

[29] Fierstra J., Sobczyk O., Battisti-Charbonney A., Mandell D.M., Poublanc J., Crawley A.P., Mikulis D.J., Duffin J., Fisher J.A. (2013). Measuring cerebrovascular reactivity: What stimulus to use?. J. Physiol..

[30] Magon S., Basso G., Farace P., Ricciardi G.K., Beltramello A., Sbarbati A. (2009). Reproducibility of BOLD signal change induced by breath holding. Neuroimage.

[31] Bright M.G., Murphy K. (2013). Reliable quantification of BOLD fMRI cerebrovascular reactivity despite poor breath-hold performance. Neuroimage.

[32] Sobczyk O., Fierstra J., Venkatraghavan L., Poublanc J., Duffin J., Fisher J.A., Mikulis D.J. (2021). Measuring Cerebrovascular Reactivity: Sixteen Avoidable Pitfalls. Front. Physiol..

[33] Sobczyk O., Battisti-Charbonney A., Fierstra J., Mandell D.M., Poublanc J., Crawley A.P., Mikulis D.J., Duffin J., Fisher J.A. (2014). A conceptual model for CO_2_-induced redistribution of cerebral blood flow with experimental confirmation using BOLD MRI. Neuroimage.

[34] Igarashi C., Okazawa H., Islam M.M., Tsujikawa T., Higashino T., Isozaki M., Kikuta K.I. (2021). Differences in Hemodynamic Alteration between Atherosclerotic Occlusive Lesions and Moyamoya Disease: A Quantitative ^15^O-PET Study. Diagnostics.

